# The Application of Organs-on-a-Chip in Dental, Oral, and Craniofacial Research

**DOI:** 10.1177/00220345221145555

**Published:** 2023-02-01

**Authors:** C. Huang, F. Sanaei, W.P.R. Verdurmen, F. Yang, W. Ji, X. F. Walboomers

**Affiliations:** 1 Department of Dentistry–Regenerative Biomaterials, Radboud Institute for Molecular Life Sciences (RIMLS), Radboud University Medical Center, Nijmegen, The Netherlands; 2 The State Key Laboratory Breeding Base of Basic Science of Stomatology (Hubei-MOST) & Key Laboratory of Oral Biomedicine Ministry of Education, School & Hospital of Stomatology, Wuhan University, Wuhan, China; 3 Department of Biochemistry, Radboud Institute for Molecular Life Sciences (RIMLS), Radboud University Medical Center, Nijmegen, The Netherlands; 4 Department of Implantology, School & Hospital of Stomatology, Wuhan University, Wuhan, China

**Keywords:** pulp biology, mucosal immunity, biofilm(s), dentin, mineralized tissue/development, odontoblast(s)

## Abstract

The current development of microfluidics-based microphysiological systems (MPSs) will rapidly lead to a paradigm shift from traditional static 2-dimensional cell cultivation towards organized tissue culture within a dynamic cellular milieu. Especially organs-on-a-chip (OoCs) can very precisely re-create the mechanical and unique anatomical structures of the oral environment. This review provides an introduction to such technology, from commonly used chip materials and fabrication methods to the application of OoC in in vitro culture. OoCs are advantageous because of their small-scaled culture environment, the highly controlled dynamic experimental conditions, and the likeness to the in vivo structure. We specifically focus on current chip designs in dental, oral, and craniofacial (DOC) research. Also, future perspectives are discussed, like model standardization and the development of integrated platforms with advanced read-out functionality. By doing so, it will be possible for OoCs to serve as an alternative for animal testing and to develop highly predictive human models for clinical experiments and even personalized medicine.

## Introduction

The phrase “health starts from the mouth” indicates that our oral and systemic health are closely interrelated. However, the unique anatomical structures within oral environment, the constant mechanical challenges, and the complex biophysics currently impede further development of in vitro research in stomatology. Microphysiological systems (MPSs) were introduced as novel in vitro culture systems with improved resemblance to tissue physiology (Ingber 2022). Static MPSs, such as self-organized organoids and microengineered tissues, have been demonstrated to recapitulate the architectural integrity of oral tissues (Gao et al. 2021). In order to further reproduce the complex oral environment, also dynamic MPSs based on microfluidics were developed and introduced in dental, oral, and craniofacial (DOC) research.

Microfluidics is the technology of processing or manipulating small amounts of fluids (~10^–9^/10^–12^ to 10^–18^ L) in micrometer-sized channels, chambers, or wells that are patterned in a microdevice referred to as a “chip” (Whitesides 2006). When (groups of) cells are assembled into the chip, the dynamic MPS generally is referred to as an organ-on-a-chip (OoC). With the application of different chip designs, cells can be organized into different natural tissue structures. Basic 1-chamber chips were used to create oral mechanical conditions for in vitro culture, for instance, in oral biofilm research, including replicating shear stress on the biofilm caused by saliva and toothbrushing action (Rath et al. 2017; Luo et al. 2019; Kristensen et al. 2020). Multifactorial and high-throughput screening on biofilms was achieved using multiarray chips, allowing for an individual niche in each well of the chip (Lam et al. 2016; Jalali et al. 2021). Parallel-chamber chips have been used to assemble tissue-specific cells into, for instance, a mucosa-on-a-chip (Rahimi et al. 2018; Ly et al. 2021), dentin-on-a-chip (Niu et al. 2019), tooth-on-a-chip (Franca et al. 2020; Rodrigues et al. 2021; Franca et al. 2022), and oral carcinoma-on-a-chip (Li et al. 2016; Liu et al. 2016). By controlling the flow of media through chambers in serially connected platforms, multiple-step events were successfully simulated, like systemic immunotoxicity and digestion.

This review summarizes the current developments and advantages of OoC models in fundamental in vitro research. Seeing the potential of the OoC technology, we anticipate a paradigm shift from traditional 2-dimensional (2D) culture to a systematic microtissue assembly within a dynamic cellular milieu. Finally, possible improvements of microfluidics approaches in DOC research are discussed.

## Introduction of OoCs

### Chip Material and Fabrication Methods

**Figure 1.**
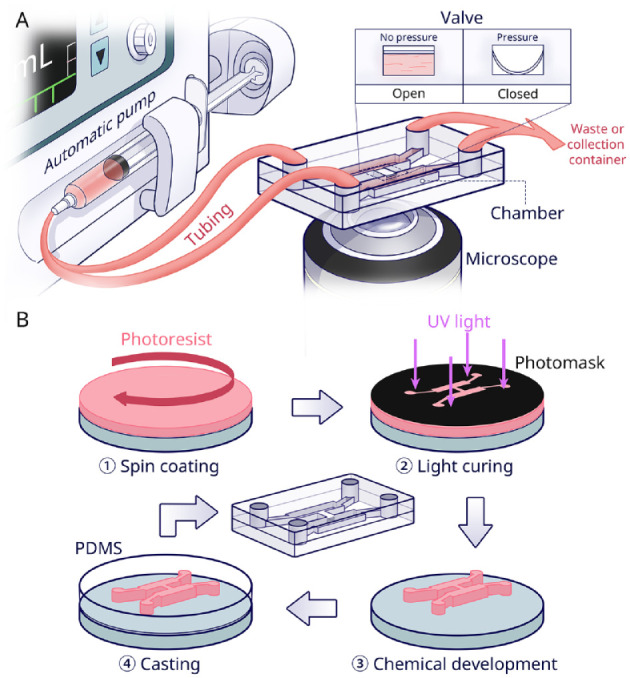
Overview of microfluidic chip technology. (**A**) Schematic of an automated microfluidic chip with monitoring microscope. The basic chip components include an inlet and outlet, cell culture chamber, and media transportation channel. A pump and valve are used for fluidic control in the chip. The valve is usually controlled by pressure. (**B**) Fabrication flow of a polydimethylsiloxane (PDMS) chip (adapted from Scott and Ali 2021). The so-called soft lithography production method, first, a photoresist material (pink), is spin-coated on a (usually silicon) substrate (gray). By UV irradiation through a photomask (black), the desired pattern is transferred onto the photoresist-coated substrate. The exposed part is subsequently cured and the non-cross-linked resist is removed. Thus, a master mold is fabricated. From the mold, PDMS casting leads to the correct microfluidic architecture. Finally, after sealing the channels and chambers by a cover, the PDMS chip is completed.

Microfluidic chips commonly contain compartments such as reservoirs, chambers, and microchannels. Moreover, there can be functional components, like valves, mixers, and pumps, which are intended to move the liquid in a determined mode (Fig. 1A).

There are various materials and microfabrication methods for production of OoCs. Using photolithography, nanometer-scale features of chips can be fabricated into silicon wafers. Nonetheless, due to the high costs, photolithographically patterned silicon is not directly used for culture but rather for the fabrication of master molds (Madou 2018). Laboratory setups then mostly use polydimethylsiloxane (PDMS) silicone rubber for the fabrication of the OoCs themselves, as explained in Figure 1B. PDMS facilitates cell culture with appropriate mechanical properties, high gas permeability, and cytocompatibility, as well as provides good optical clarity and low autofluorescence for microscopical observation (Nge et al. 2013). However, there are also shortcomings in the use of PDMS chip for quantitative experiments, including nonspecific adsorption of proteins or small molecules, surface hydrophobicity, and liquid evaporation (Ren et al. 2013).

Thermoplastic chips are alternatives for quantitative experiments. Poly-methylmethacrylate chips fabricated by micromilling have been used as tooth-on-a-chip or skin-on-a-chip for toxicological applications (Sriram et al. 2018; Hu et al. 2022). In industrial settings, injection molding and embossing are popular to decrease fabrication cost and achieve upscalability (Low et al. 2021). Three-dimensional (3D) printing can be used to fabricate chips with complex structures in a single step. However, in printing generally, it is challenging to fabricate features smaller than 200 µm with high shape fidelity (Urrios et al. 2016). In addition, not all biocompatible materials are printable, such as poly-methylmethacrylate and polycarbonate (Naderi et al. 2019; Low et al. 2021). For a more comprehensive description of alternative materials and fabrication processes, we refer the reader to specialized reviews (Nielsen et al. 2020; Scott and Ali 2021).

### Advantages of OoCs in In Vitro Culture

**Figure 2.**
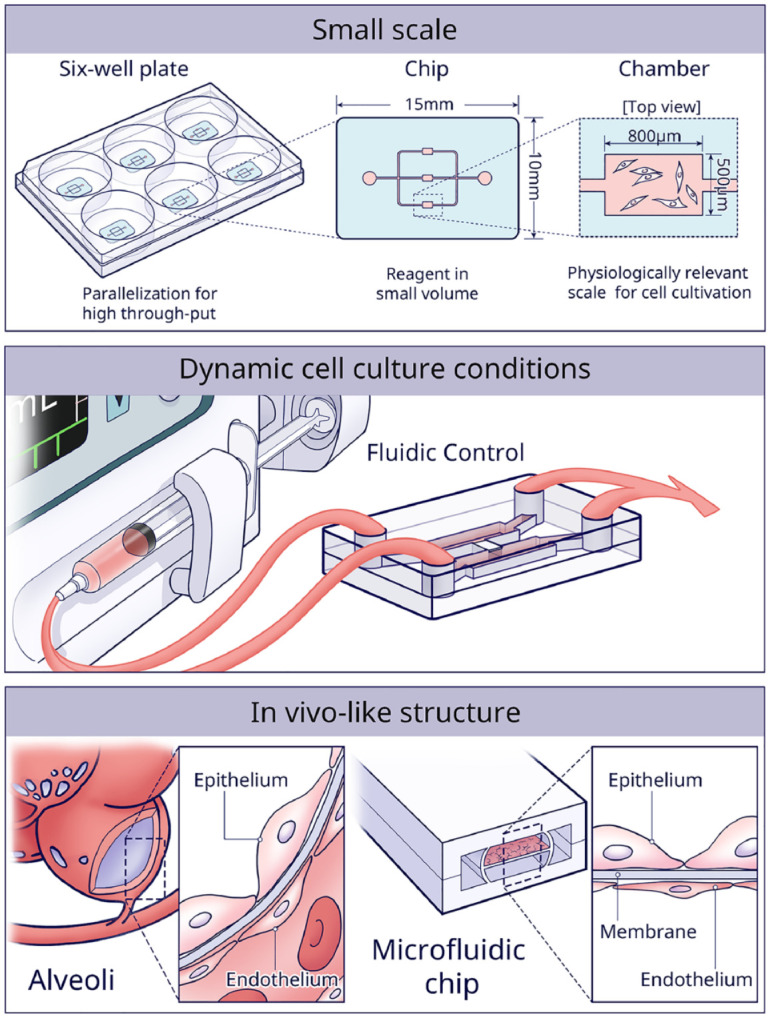
The 3 main advantages of using microfluidic chips for in vitro culture are the small scale of the models, the high control over dynamic cell culture conditions, and the possibility to efficiently construct in vivo–like structures.

Regardless of fabrication and material choice, the OoC possesses clear advantages over the conventional macroscale 2D cell culture technique (El-Ali et al. 2006; Mehling and Tay 2014)—namely, 1) the small scale of the model, 2) the considerable control over dynamic experimental conditions, and 3) the likeness to the in vivo structure (Fig. 2). All 3 aspects will be detailed in the following paragraphs.

First, micrometer-sized culture chambers are not only cost-effective but especially provide a more physiologically relevant scale to maintain cellular phenotype and function (Ingber 2022). For instance, when 2 different bacteria were cocultured in microscale chambers, an exclusion zone around the perimeter of 1 bacteria colony was formed, where the other type did not grow. However, the same phenomenon did not occur in traditional macroscopic media (Jalali et al. 2021). The results indicated that the small volume enhanced the quorum sensing and competition, similar to the in vivo situation. Another evident advantage of small-scale culture chambers is parallelization for high-throughput experiments. For instance, a microfluidic platform yielding ~107 salivary gland mimetics showed great potential for high-content drug screening (Song et al. 2021).

Second, the continuous supply of fresh media provides cells with a stable environment and shields cells from biochemical changes, such as waste accumulation or calcium/phosphate imbalance (Atif et al. 2021). Various studies have shown that flow-induced shear and mechanical stresses can simulate the in vivo mechanical cues, which of course are a key determinant of cell behavior (LeGoff and Lecuit 2015). For example, with shear stress simulating the orthodontic force, cementocytes showed greater potential in bone remodeling than osteocytes (Xie et al. 2018). Another study used a peristaltic pump (300 μL/min flowrate) to mimic the mechanical environment of periodontal ligament–alveolar bone interface (Vurat et al. 2022). In addition, a unidirectionally gradient flow in gingival crevice was simulated by creating difference in hydrostatic pressure between side channels (Makkar et al. 2022). Furthermore, microfluidic chips with sequentially timed fluid control can deliver precise spatiotemporal biochemical signals from cytokines (Ai et al. 2018). Conventionally, supplemented or conditioned media are used, but many media changes would limit such an approach.

Third, OoC is advantageous to control cell assembly in 3D native tissue-like structures. The first OoC created in 2010 consisted of a breathing-lung-on-a-chip (Huh et al. 2010). The structure of the alveolar–capillary interface was mimicked by seeding epithelial and endothelial cells on opposite sides of a PDMS membrane. A vacuum was applied to induce stretching of the membrane, which re-created physiological breathing movement. Inspired by this work, the gingival epithelium–capillary interface was re-created (Jin et al. 2022). During the past decade, many other OoCs have been developed in biological research, such as bone-on-a-chip, liver-on-a-chip, kidney-on-a-chip, gut-on-a-chip, and cardiac muscle/heart-on-a-chip (Ahadian et al. 2018). In this review, we will focus specifically on chips dedicated to DOC research.

## OoCs in DOC Research

As mentioned above, the inherent advantages of OoCs are promising to address the 2 main difficulties in current DOC research: 1) to simulate the multifactorial oral environment (e.g., dynamic salivary flow, temperature change, pH fluctuation) and 2) to mimic tissue interfaces (e.g., biofilm–tooth, dentin–pulp, biomaterial–mucosa). When trying to categorize DOC models, it becomes evident that the overall design of the chip determines the function and thus the application. In the following sections, the chips in current oral research are organized into 4 major categories: the 1-chamber, the multiarray, the parallel-chamber, and the serial-chamber designs. Thereafter, the application of each design is summarized.

### One-Chamber Design

The 1-chamber chip forms the most basic design, consisting of a single culture chamber linked to channels for fluid transport. With this design, diverse mechanical oral environments can be simulated. For instance, the mechanical stress caused by saliva flow is inescapable in the oral environment and tightly related to the formation and characteristics of a biofilm. To investigate the accumulation of biofilms on an implant surface, a hibernation mode of saliva flow was simulated by setting the flow speed to 100 μL/min (Rath et al. 2017). Five oral commensal and periodontopathogenic bacteria reproducibly formed a biofilm on the titanium surface, as evidenced by 3D reconstruction with confocal microscopy. The presented system was easily applicable to other materials of interest too.

The dynamic pH changes of oral biofilms could also be monitored on 1-chamber chips (Gashti et al. 2016; Kristensen et al. 2020). To study the impact of saliva flow on biofilms’ pH change, a stimulating flow velocity of 5 mm/min was used (Kristensen et al. 2020). In static culture, the pH in the top layer of the biofilms tended to be lower than at the bottom. However, under saliva flow, the vertical gradients of pH were reversed and even rose to slightly alkaline values. The opposite pH profiles observed between the 2 conditions confirmed the significance of having flow in biofilm studies.

In addition, the 1-chamber design was used to study an individual’s oral health care routine (Luo et al. 2019). In a model system developed by Luo et al. (2019), toothbrushing treatment automatically occurred at both 8 h (morning) and 18 h (evening). A shear stress on the biofilm mimicking the brushing was achieved by setting flow at 2.0 dynes/cm^2^ for 2 min. Image analysis software was applied to quantify biofilm architecture. Results showed that stannous ions, as present in toothpaste, resulted in decreased biofilm volume, surface area, number of objects, and connectivity, all in a dose-responsive manner.

### Multiarray Design

In a multiarray chip, multiple chambers of the same size are connected by channels and arranged in a matrix. The chambers are functional as the cell culture wells. By producing different conditions in the individual chambers, this design is mainly used for high-throughput screening.

**Figure 3.**
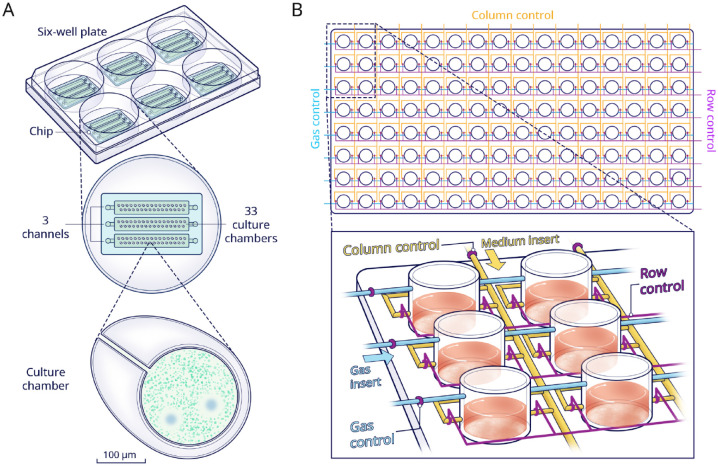
Multiarray chips. (**A**) Schematic view of a multiarray chip with 99 chambers distributed in 3 independent channels evenly. By adding 1 chip to each well of a 6-well culture plate, 18 different conditions are supported. Lower panel shows that bacterial exclusion zones were assessed in the small-scale chambers when coculturing different bacteria. (**B**) Schematic of a high-throughput platform with 128 chambers (8 rows × 16 columns). The 8 chambers in each column are connected by media-transporting channels (yellow). The column control and row valves (purple) are intended to control the media insertion in each individual chamber. In addition, dissolved oxygen conditions can be controlled through a gas insert (blue) in combination with control valves.

Jalali et al. (2021) developed a chip with 99 chambers to investigate the interplay among multiple bacterial strains (Fig. 3A). In this study, they mixed 5 strains of *Actinomyces* and 3 strains of *Schaalia* with 7 strains of *Streptococcus*. Among those 56 strain combinations, the strain of *Actinomyces graevenitzii* with *Streptococcus cristatus* and *Streptococcus salivarius* showed the formation of bacterial exclusion zones. Exclusion zones also occurred in the coculture of *A. graevenitzii* and *Staphylococcus aureus*. These results indicated that specific interaction was only triggered by the *A. graevenitzii* nearby. Although this design requires manual handling and is incompatible with cell-staining assays, it provided a simpler and more cost-effective method compared to well plates.

A multifactorial environment can also be achieved through a design with multiplexed channels and valves. A device, consisting of 8 rows × 16 columns of culture chambers, was developed by Lam et al. (2016) (Fig. 3B). Media with 16 different sucrose concentrations could be injected through liquid inlets into a selected chamber at any time point. Furthermore, the 8 rows were grouped into independent conditions of dissolved oxygen. Thus, 128 different profiles could be provided for parallel cultivation and analyses. Fluorescence in situ hybridization was implemented to identify, in real time, biofilm morphology, colonization density, and spatial arrangement. Results showed that the coverage ratios of *Streptococci*, *Fusobacterium nucleatum*, and *A. graevenitzii* in the biofilm were comparable to the in vivo ratio. It was further demonstrated that sucrose ≥1% (w/w) promoted the attachment of streptococci and facilitated further cocolonization with *F. nucleatum*. Finally, it was indicated that aerobic streptococci were capable of consuming the available oxygen, thus creating local hypoxia for the anaerobic *F. nucleatum* to survive.

### Parallel-Chamber Design

The parallel-chamber chip is mostly used as a scaffold to simulate natural tissue architecture, in order to investigate pathophysiological processes. In this design, 2 or more parallel chambers are connected vertically or horizontally with a variety of structures in between, like pores, membranes, or tubes. There is elaborate literature on this approach in oral research, mucosa-on-a-chip, dentin-on-a-chip, tooth-on-a-chip, and so on, which all will be reviewed hereafter.

**Figure 4.**
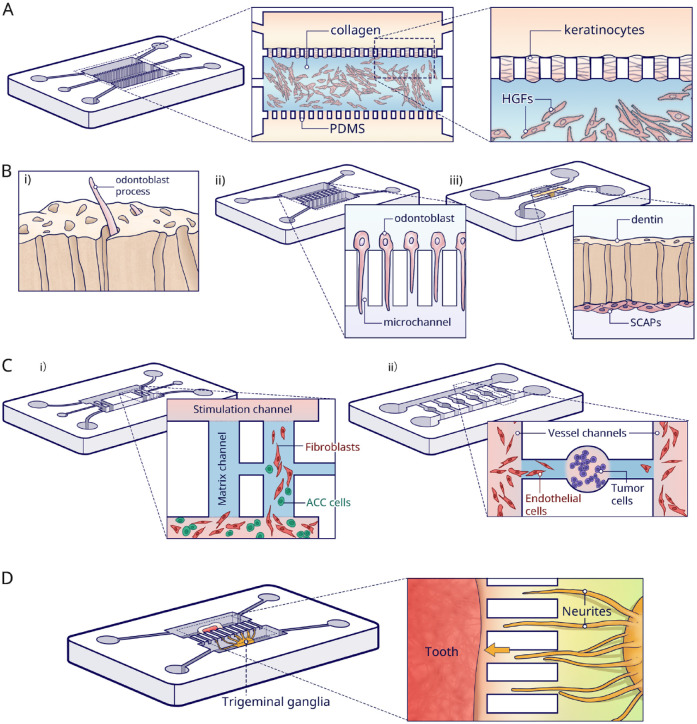
Parallel-chamber chips. (**A**) In the mucosa-on-a-chip, fibroblasts were seeded in collagen in the central channel, and keratinocytes were grown on top. The upper channel was used for the insertion of dental materials, and the bottom channel was for the media. (**B**) Dentin-on-a-chip and tooth-on-a-chip models. i) Illustration of a functional dentin–pulp complex. ii) For the dentin-on-a-chip system, microchannels were made to induce odontoblast processes. iii) Tooth-on-a-chip with a native dentin disc inserted in between 2 channels. Pulp cells were seeded in 1 channel and adhered to the dentin. The opposite channel was used to provide exogenous oral components. (**C**) Adenoid cystic carcinoma (ACC)-on-a-chip model. i) ACC-related fibroblasts were cocultured with ACC cells in the bottom channel. The media in the upper channel induced cells to migrate through the vertical channel. The invasion pattern in the vertical channel showed that fibroblasts (red) localized at the invasion front and ACC cells (green) following behind. ii) Another ACC-on-a-chip for the investigation of tumor-induced angiogenesis. The ACC cells were seeded in the round chamber and the Human umbilical vein endothelial cells (HUVECs) were seeded in the vessel channels. The angiogenesis was then tracked in the side channels. (**D**) Tooth innervation on a microfluidic chip. Trigeminal ganglia and tooth tissue were cultured in parallel chambers in different media. The neurites (yellow) are growing toward the tooth (red) through the microgrooves.

Rahimi et al. (2018) developed an oral mucosa-on-a-chip with histologically correctly configured epithelial and fibrous layers (Fig. 4A). Fibroblasts suspended in collagen were loaded in the central channel, and subsequently keratinocytes were seeded between pillars on the apical layer. With apical-basal geometry and good transparency, the mucosa-on-a-chip allowed for convenient and precise tracking of responses to dental biomaterials and oral bacteria. Microscopical observation was used for readout. The results proved that the mucosa-on-a-chip was more sensitive in assessing cell viability than well-plate cultures when exposed to a common dental material like 2-hydroxyethyl methacrylate, especially at lower doses (Ly et al. 2021). However, the contraction of collagen matrix limited the culture period and resulted in poor epithelium stratification. Moreover, the pillars led to the formation of a discontinuous epithelial layer. Finally, an in vivo comparison would be needed to verify and further improve the physiological relevance.

Likewise, dentin-on-a-chip has also been described. In vivo, odontoblasts have their cell bodies in the periphery of the dental pulp, and cytoplasmic projections grow toward the dentin tubules (Fig. 4Bi). Projections play an important role in the transduction of external stimuli. However, such unique morphological characteristics disappear in traditional culture (Alvarez et al. 2017). Niu et al. (2019) successfully replicated the dentinal architecture (Fig. 4Bii). The used dentin-on-a-chip device contained 2 parallel chambers that were connected by multiple 2-μm-wide microchannels simulating the tubules. Hydrostatic pressure was applied to drive the odontoblasts from one chamber to the opposite. Subsequently, odontoblast projections were induced, simply because the small width of the microchannels constrained the migration of the whole odontoblast cell body through the channel. Immunofluorescence demonstrated that cells presented a similar morphology to odontoblasts in vivo, and moreover, the processes expressed the odontoblast marker AQP4. However, using PDMS microchannels rather than real dentin in chip largely oversimplified the dentin–pulp environment, which hinders further application of this system in dental biomaterial testing and investigation of the dentinal repair process.

In an actual tooth, the dental pulp and surrounding dentin together are regarded as a functional complex responsible for all vital responses. The first tooth-on-a-chip model consists of 2 parallel channels (Franca et al. 2020) (Fig. 4Biii). One channel represented the pulp cell side, and the other side was a cavity in which it was possible to provide exogenous oral components (i.e., bacteria, dental materials, and saliva flow). A native dentin disc was inserted between these 2 channels. This tooth-on-a-chip was evaluated as a testing platform replicating the step-by-step process of a restorative treatment. Materials such as phosphoric acid, dental adhesive systems, and monomers were tested for cytotoxicity, cell morphology, and metabolic activity in comparison to conventional control models. With dentin as a semipermeable barrier, the pulp cells presented consistently higher metabolic activity and were less susceptible to injuries than those exposed directly to the test materials. More recently, this same tooth-on-a-chip was also used to investigate the early interplay of calcium silicate cement with dental pulp stem cells (DPSCs). The model verified that such events correlated with pH variations and growth factor release (Rodrigues et al. 2021). Furthermore, a biomaterial–biofilm–dentin interface was established with *Streptococcus mutans*, to test the antimicrobial capacity of calcium silicate cement. Results suggested that calcium silicate indeed can disrupt the structural integrity of a biofilm and simultaneously kill bacteria within. However, it was technically challenging to assemble dentin disc with the cover slip. In this tooth-on-a-chip, assembly was done by slightly applying pressure yet without sealing, which is prone to leakage. This critical step may be the reason why static culture conditions were chosen, instead of including saliva flow and/or blood flow in dental pulp. Recently, the physiological blood flow was simulated in a vertical bilayer chip, where a dentin disc was clamped above a rhomboid-shaped culturing chamber for DPSCs, with a flow channel in between (Hu et al. 2022). In this way, the part of the flow from the inlet toward the disc/cells could be analyzed and serve as the internal control and the section of the flow thereafter as the experimental situation.

Besides representing tissue structure, the parallel design is beneficial to mimic pathological processes in vitro, like the invasion and metastasis of adenoid cystic carcinoma (ACC) (Liu et al. 2010; Kong et al. 2016; Kong et al. 2018). ACC-on-a-chip was built to investigate the invasion pattern of salivary gland ACC (Li et al. 2016) (Fig. 4Ci). Carcinoma-associated fibroblasts and ACC cells were cocultured in a channel with serum-free media, whereas 20% serum was inserted into the opposite stimulation channel. In this way, the mixed cells migrated to the opposite channel through the linking channels. The model indicated that the pattern of ACC invasion was that of carcinoma-associated fibroblasts localizing at the invasion front, whereas the ACC cells followed the track. Nevertheless, using Matrigel as a substitute for extracellular matrix (ECM) is controversial. First, the composition of Matrigel is not exactly defined, which may lead to batch-to-batch variability in the results. Also, linkage of 2 matrix channels by 1 narrow inserting channel makes it difficult to accurately control the matrix injection and maintenance.

Furthermore, paralleled chips were used to investigate the angiogenesis process in dental pulp regeneration (Zhang et al. 2022) and oral tumor (Liu et al. 2016). On a tumor-induced angiogenesis chip, each tumor unit consisted of a cell culture chamber to mimic the primary tumor, combined with 2 side branches linked to bilateral vessel channels separately (Fig. 4Cii). The tumor-induced angiogenic process was monitored at several time points. The results showed both the invasion distance and area induced by ACC were significantly lower than by a squamous cell carcinoma, which were consistent with the animal models.

Finally, this design enables the investigation of physical interaction between 2 organs. Pagella et al. (2014) seeded tooth tissue in 1 compartment and trigeminal ganglion in a parallel compartment. These 2 compartments were linked by multiple microgrooves (Fig. 4D). Hence, the in vivo innervation process of the embryonic tooth germ or postnatal pulp tissue was successfully reproduced on the chip while coculturing ganglia and tooth germs in their specific culture media (Pagella et al. 2014). The same result was not obtainable by conventional direct coculturing, which resulted in degeneration in a short period and in markedly different neuronal behavior. The same design was used to investigate the neurotrophic effects of DPSCs on trigeminal (Pagella, Miran, et al. 2020) and ameloblastoma innervation (Pagella, Caton, et al. 2020).

### Serial-Chamber Design

By connecting various organ or tissue models, each in an individual chamber or set of chambers, into an interconnected network, elaborate chip layouts enable one to emulate the relevant physiological process in vitro, like an immune system or a digestive system.

**Figure 5.**
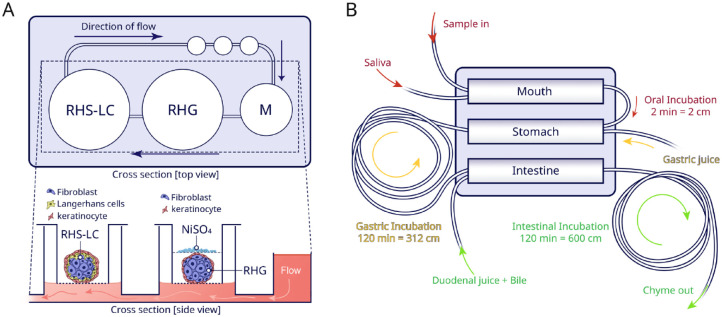
Serial-chamber chips. (**A**) Schematic top and cross-section view of the immunosystem-on-a-chip. Media (M) flow direction was from reconstructed human gingiva (RHG) to reconstructed human skin with Langerhans cells (RHS-LC). After culture in the chip, the RHG were exposed to nickel sulfate (NiSO_4_) and then used for the analysis. (**B**) Representation of the digestive-track-on-chip. This chip consists of a mouth, stomach, and intestine chamber. In each chamber, the flow was mixed with different digestive juices in physiologically relevant ratios. Connected to the outlet of each compartment, tubing loops were used for incubation. Samples can be collected at different time points in these loops to closely assess the whole digestive progress.

An immunosystem-on-a-chip (Fig. 5A) was developed to study systemic immunotoxic events involving distant organs rather than investigating local events in a single tissue or organ (Koning et al. 2021). To represent inflammation, activated by exposure of gingiva to nickel, 2 cell culture chambers were set in a closed circuit on a chip. This particular study combined OoC with organoid technology (for detailed review on organoids; see Clevers 2016). Gingiva and skin organoids with immune (Langerhans) cells were constructed and settled in culture chambers separately. After exposing the gingiva to nickel sulfate (NiSO_4_), flow was applied to the skin part. Quantitative RT-PCR and immunofluorescence showed that nickel exposure of gingiva resulted in increased activation of Langerhans cells in the skin organoids.

A miniaturized digestive-tract-on-chip was fabricated by means of enzymatic reactions (de Haan et al. 2019) (Fig. 5B). On this chip, 3 compartments were coupled in series to mimic the mouth, stomach, and intestine, respectively. The model demonstrated enzymatic functionality, through assessment of fluorescent compounds. Even the bioavailability of orally consumed drugs could be investigated in the digestive system (de Haan et al. 2021).

## Future Directions

As a new class of research tools, the microfluidic approach has been widely applied in biomedical research and now also has come to prominence in DOC research. However, as with any novel technique, there are several shortcomings and challenges that need consideration.

First, substantial efforts are needed to achieve model standardization, which is one of the key determinants for general acceptance. So far, only few in vitro models have been introduced in DOC, but the variability between the same tissue models from different research groups is considerable (Table). For example, to study the pulp response toward dental materials, 1 model used a monolayer of predifferentiated stem cells from the apical papilla (SCAPs) (Franca et al. 2020), while the only comparable study selected a 3D ECM with DPSCs but without predifferentiation (Rodrigues et al. 2021). Instead of constructing laboratory-specific OoCs, it would be advisable to invest more efforts in collaboration to develop uniform constructing protocols upon the early development of such a complex technology. The same argument goes for experimental conditions, for instance, with 1 study mentioning flow in volume (µL/min), the other in velocity (µm/min), and a third describing forces (dynes/cm^2^).

**Table.** Summary of Various In Vitro Model Construction Parameters and the Potential Applications.

Aim	Chip Design	Material and Fabrication	Combined Biomaterials	Cell/Bacterial Type	Culture Parameters	Data Collection Methods and Contents	Future Applications	Reference
To investigate accumulation of biofilms on (titanium) implant surface	One-chamber	Polyaryletherketone platform assembly with multiple materials	○ None	○ *Streptococcus gordonii* ○ *Streptococcus oralis* ○ *Streptococcus salivarius* ○ *Porphyromonas gingivalis* ○ *Aggregatibacter actinomycetemcomitans*	○ Media: each species in appropriate bacterial media ○ Flow rate: 100 μL/min	○ Confocal microscopy with live–dead staining ● Side view reconstruction of biofilm ● Mean height of biofilm	Test model for the antibacterial effect of dental materials	(Rath et al. 2017)
To quantify the architecture of oral biofilms in antibiofilm interventions (Sn^2+^) with an image analysis program	One-chamber	PDMS for soft lithography and assembly with multiwell and cover glass	○ None	○ Bacteria from healthy volunteers	○ Media: saliva from healthy individuals ○ Flow rate: 2 dynes/cm^2^	○ Confocal microscopy with live–dead staining ● Biovolume ● Number of objects ● Surface area ● Fluffiness ● Connectivity ● Convex hull porosity ○ Viability	In vitro model for testing antimicrobial reagents	(Luo et al. 2019)
To study the impact of stimulated saliva flow on pH changes in dental biofilms	One-chamber	Resin 3-dimensional printing	○ None	○ Biofilm from a healthy volunteer	○ Media: stimulated saliva samples from participant ○ Flow rate: 5 mm/min	○ Confocal microscopy with pH-sensitive dye ● pH measurements	In vitro model for pH measurements in in situ–grown biofilm	(Kristensen et al. 2020)
To investigate the chemical and hydrodynamic affections on pH changes of oral biofilms	One-chamber	PDMS for soft lithography and assembly with a pH-sensor-coated glass substrate	○ None	○ *Streptococcus salivarius*	○ Media: unbuffered modified LB growth medium ○ Flow rate: 0 mL/h; 0.1 mL/h; 0.3 mL/h	○ Confocal laser scanning microscope with luminescence ● pH-sensitive sensors	In vitro model for localized acidification at the oral biofilm	(Gashti et al. 2016)
To assess the impact of the dentin barrier and permeated silver diamine fluoride on cells	One-chamber with a dentin disc above	Thermally bonding poly(methyl methacrylate) sheets fabricated by micromilling	○ None	○ DPSCs	○ Media: DMEM ○ Flow rate: 1.5 μL/min	○ Confocal microscopy with live–dead staining	Dentin barrier test model for dental materials	(Hu et al. 2022)
To study the process of endothelialization and angiogenic sprouting	One -chamber with varying taper	PDMS for soft lithography and assembly with a tapered chamber built-in GelMA	○ GelMA ○ Angio-Proteomie	○ SCAPs ○ HUVECs	○ Media: EGM for coculture	○ Confocal microscopy with HUVECs-GFP ● Angiogenic sprouting	Potential strategy for prevascularized pulp tissue construction	(Qi et al. 2021)
To characterize dynamic interactions between oral bacteria	Multiarray	PDMS soft lithography	○ None	○ *Actinomyces* species (5) ○ *Schaalia* species (3) ○ *Streptococcus* species (7)	○ Media: IMDM with FBS	○ Automated microscope ● Bacterial exclusion area	Device for screening the interaction of multiple bacterial species at microscale	(Jalali et al. 2021)
To investigate the growth of *Streptococci* species and *Fusobacterium nucleatum* in biofilm under different dissolved gas and sucrose concentrations	Multiarray	PDMS soft lithography	○ None	○ Biofilm collected from human oral cavity	○ Media: nutritional analogue of saliva	○ Automated microscope with a motorized xyz-stage ○ Fluorescence in situ hybridization ● Biofilm thickness ● Viable–dead cell ratio ● Spatial distribution of multiple bacteria	Device for high-throughput and quantitative analysis of dental bacteria under different combinations of microenvironmental factors	(Lam et al. 2016)
To mimic the gingival crevicular environment with host-microbial colonization in health or disease	Parallel	PDMS soft lithography	○ Fibrin gel	○ Human gingival fibroblasts ○ *Streptococcus oralis* ○ *Fusobacterium nucleatum*	○ Media: Opti-MEM I reduced serum medium	○ ELISA for detection of inflammatory factors ○ Lactate dehydrogenase for cocultured system viability ○ Confocal microscopy ● Live–dead staining for cell viability ● Fluorescent dye for interstitial flow perfusion ● Immunostaining for cell morphology ● Fluorescent labeling for bacteria distribution	In vitro gingival crevice model for periodontitis study	(Makkar et al. 2022)
To develop a valid model for periodontal soft tissue	Parallel	PDMS for soft lithography, assembly with a porous polyester membrane	○ 3-Glycidoxypropyltrimethoxysilane ○ 3-Aminopropyltriethoxysilane	○ HUVECs ○ Human gingival epithelial cells	○ Media: ● Keratinocyte growth medium for gingival epithelial cells ● EBM-2 for HUVECs ○ Inflammation treatment: LPS or TNF-α; inhibitor: PDTC	○ Confocal microscopy ● Live–dead staining for cell viability ● Celltracker for cell distribution ● Immunostaining for interface junction ○ ELISA for detection of inflammatory factors	In vitro periodontal model for drug assays and for functional investigation	(Jin et al. 2022)
To assess cytotoxicity of dental material (HEMA) and *Streptococcus mutans* on mucosa	Parallel	PDMS soft lithography	○ Collagen I	○ *Two immortalized human cell lines* ● *Keratinocytes (Gie)* ● *Fibroblasts (HGF)* ○ *Streptococcus mutans*	○ Fibroblasts were mixed with collagen ○ Keratinocytes were seeded on top of fibroblast layer ○ Media: Prigrow III/IV media (1:1)	○ Epifluorescence microscopy ○ Phase contrast microscopy ● Actin and nuclei staining for cell morphology and organization ● Live–dead staining for cell viability ○ Transepithelial electrical resistance contrast to detect epithelial barrier function	In vitro model to study mucosal interaction with bacteria and biomaterials	(Rahimi et al. 2018)
To assess the oral mucosa response to different concentration of dental material (HEMA)	Parallel	PDMS soft lithography	○ Collagen I	○ Two immortalized human cell lines ● Keratinocytes (Gie) ● Fibroblasts (HGF)	○ Media: Prigrow III/IV media (1:1)	○ Epifluorescence microscopy ○ Confocal microscopy ● Cytoskeleton and nuclei staining for cell organization and voids area calculation ● Live–dead staining for cell viability	In vitro model to study mucosal interactions with biomaterials	(Ly et al. 2021)
To investigate the suitable size of microchannels for inducing odontoblast processes	Parallel	PDMS soft lithography	○ Collagen I	○ Odontoblast cell line MDPC-23	○ Media: DMEM with FBS	○ Microscopy ● Cytoskeleton and nuclei staining for cell morphology and position ● Biomarker staining for cell function	Model for investigating the physiology and pathology of odontoblast processes	(Niu et al. 2019)
To develop a functional pulp–dentin model for dental material testing	Parallel	PDMS soft lithography	○ None	○ SCAPs	○ Media: -MEM with embryonic stem cell FBS ○ Predifferentiated 10 d before seeding in chip	○ Confocal microscopy ● Actin filaments and nuclei staining for cell morphology and proliferation ● Live-cell imaging for cell position, response to materials ● DNA dye staining for viability ● Gelatinolytic activity ○ Metabolic activity	Dentin-pulp test model for dental materials	(Franca et al. 2020)
To investigate antibiotic ability of calcium silicate and interactions with pulp	Parallel	PDMS soft lithography	○ Collagen I	○ Human dental pulp stem cells	○ Media: -MEM with FBS ○ 3-dimensional culture in collagen	○ Confocal microscopy ● Actin and nuclei staining for cell morphology ● Live–dead staining for cell viability ○ Measurement of pH and TGF-β in solution	Biofilm–dentin–pulp model to test dental materials and investigation of mechanism	(Rodrigues et al. 2021)
To study the role of carcinoma-associated fibroblasts in the invasion of ACCs	Parallel	PDMS soft lithography	○ Matrigel	○ Primary cells: fibroblasts from ACC patients ○ Cell lines: ACC cells (SACC-LM and SACC-83)	○ Directly cocultured fibroblasts with ACC cells ○ Media: DMEM/F12	○ Microscopy ● Cell tracker or liner for assessment of cell invasion	In vitro model to track cancer progress	(Li et al. 2016)
To explore the signaling mechanisms that recruit dental stem cells in angiogenesis	Parallel	Commercial chips from AIM Biotech	○ Fibrin gel	○ HUVECs ○ Stem cells from human exfoliated deciduous teeth	○ Media: ● Endothelial cell medium with FBS for HUVEC ● MEM with FBS for dental stem cells	○ Confocal laser scanning microscope ● Immunostaining of cell markers for recruitment and distribution assessment of dental stem cells around nascent vessels ● Fluorescent labeling dextran for vessel permeability assay	In vitro model for investigation of multistep process of angiogenesis in dental pulp regeneration	(Zhang et al. 2022)
To reproduce oral cancer–induced angiogenesis and evaluate the effect of antiangiogenic drugs	Parallel	PDMS soft lithography	○ Cultrex Basement Membrane Extract	○ Cell lines ● HUVEC ● ACC-M ● UM-SCC6	○ Media: ● Endothelial cell media with FBS for HUVEC ● MEM with FBS for ACC-M ● DMEM/high glucose with FBS for UM-SCC6	○ Microscopy ● Actin and nuclei staining for cell morphology, invasion distance, and area ● Angiogenesis biomarker staining to assess capillary-like structures	○ Oral cancer model to study the angiogenesis and drug test	(Liu et al. 2016)
To study the behavior of neurons during the tooth germ development	Parallel	PDMS soft lithography	○ Poly-D-lysine ○ Laminin	○ Trigeminal ganglia from embryonic mouse (days 15.5–16.5) ○ Incisor tooth germs from embryonic mouse (day 15.5) ○ Molar tooth germs from embryonic mouse (day 17.5) and postnatal pups (day 5)	○ Tissue culture ● Trigeminal ganglia in Neurobasal media ● Tooth germ in high glucose DMEM with FBS	○ Microscopy ● Biomarker staining of neurofilament, β-tubulin to show the interaction of neurite with tooth germ ● Immunohistochemistry ○ Interaction of neurite with tooth germ	A predictive platform for studying innervation process in orofacial tissues and organs	(Pagella et al. 2014)
To study immunoreaction of skin/oral mucosa after exposure to metals	Serial	PDMS for soft lithography and assembly with polycarbonate and cover glass	○ None	○ Skin and gingiva fibroblasts and keratinocytes from healthy donors ○ Langerhans cells cell line MUTZ-LCs	○ Organoid culture ● Gingival fibroblasts and keratinocytes were constructed to gingiva organoid ● Skin fibroblasts, MUTZ-LCs, and keratinocytes were constructed to skin organoid ○ Media: DMEM/Ham’s F-12 (3:1) ○ Pulsatile flow at 0.5 Hz and 500 mBar	○ Measurements of lactate dehydrogenase, lactate, and glucose in supernatant to reflect model stability ○ Detection of nickel ions and interleukins in supernatant ○ Quantitative RT-PCR and biomarker staining for Langerhans cell activation	Investigation of systemic immunotoxicity in a multiorgan setting	(Koning et al. 2021)
To develop a functional digestion model	Serial	PDMS soft lithography	○ None	○ None	○ Samples continuously mixed with artificial digestive juices	○ Microscopy ● Separate enzymatic assays of “mouth,” “stomach,” and “intestine” with specific fluorescent substrates and enzymes ● pH in each room was detected by fluorescein ○ SDS-PAGE to detect the whole digestion process of lactoferrin	In vitro model to study the bioavailability of orally administered compounds	(de Haan et al. 2019; de Haan et al. 2021)

α-MEM, α–modified minimal essential medium; DMEM, Dulbecco’s modified Eagle’s medium; DPSC, dental pulp stem cell; EGM, endothelial cell growth medium; ELISA, enzyme-linked immunosorbent assay; FBS, fetal bovine serum; GFP, green fluorescent protein; HEMA, Hydroxyethylmethacrylate; HUVEC, Human umbilical vein endothelial cells; IMDM, Iscove’s modified Dulbecco’s medium; LPS, lipopolysaccharide; PDMS, polydimethylsiloxane; PDTC, Pyrrolidinedithiocarbamic acid; SCAP, Stem Cells From the Apical Papilla; SDS-PAGE, sodium dodecyl sulfate polyacrylamide gel electrophoresis; TGF-β, transforming growth factor–β; TNF-α, tumor necrosis factor–α.

A second big breakthrough would be further developing 3D cultures in chip models. Matrigel and collagen/gelatin-based hydrogels are common materials integrated in microfluidic devices to mimic extracellular matrix and capable of orchestrating cell behavior and communication (Karamanos et al. 2021). Two effective methods to include a cell-laden hydrogel are by the capillary action of the channels, or simply by direct injection, which has been successfully applied in mucosa-on-a-chip (Rahimi et al. 2018; Ly et al. 2021). Sacrificial molding is sometimes used to fabricate hollow dental root structure in a hydrogel housed in a chip chamber (Qi et al. 2021). To develop physiologically correct complex structures on chip, future strategies should combine OoCs with other tissue engineering technologies, like organoids (Koning et al. 2021), micromimetics, and 3D bioprinting (Vurat et al. 2022). For instance, recently, miniaturized oral mucosa equivalents were integrated within a microfluidic chip to evaluate the permeation of dental anesthetics (Muniraj et al. 2020). Likewise, 3D bioprinting has been successfully used to introduce blood vessels in cancer cell–laden hydrogel on a chip (Cao et al. 2019). A similar technique could also be adapted to achieve vascularization in oral OoCs in the future.

A third advance would be the establishment of multiple and synchronous monitoring on OoCs without stopping the experiments, thus compensating for the shortcomings of sample extraction and insufficient amount of sample as often occurs in conventional biological assays. Various sensors enable real-time and quantitative measurements of a diverse array of cell function in situ, such as detection of the biochemical factors in the media, the integrity of barrier tissue, or the electrical activity in cells (Noh et al. 2011). Even though mucosa integrity was detected on the mucosa-on-a-chip (Rahimi et al. 2018), the current real-time monitoring of cell morphology and behavior in oral models largely relies on direct imaging by cell staining and microscopy. The incorporation of diverse sensors in OoC is necessary to collect dynamic and quantitative information during biological processes.

Finally, OoC technology holds great promise as a complementary technology to animal experimentation (Staubli et al. 2019; Wilkinson 2019), as an effective tool for the implementation of the 3R principle (i.e., Reduction, Refinement, and Replacement) (Hubrecht and Carter 2019). For instance, OoC systems can enable a superior a priori design of experiments and therefore reduce the number of animal trials with statistically insignificant results (Ingber 2022). In addition, as compared to animal models, OoC systems are advantageous in providing predictive models for human-specific physiological and pathophysiological studies (Low et al. 2021). Furthermore, being able to include patient-derived cells in OoC opens a huge potential in drug development for rare diseases, clinical experiments, and even transition from one-size-fits-all therapies to personalized medicine approaches (Ingber 2022).

## Conclusions

OoC is a very promising emerging technology bringing dynamic biomimicking microenvironments and 3D tissue architecture to in vitro cell culture. Application-specific chips have been designed for the exploration of a diversity of oral physiological and pathological processes, including the growth of biofilms, reactions of mucosa and teeth to dental materials, development of oral tumors, and tooth innervation. Furthermore, multiple-step models have been developed to study the immunotoxicity of exposed gingiva and the digestive process. In the future, standardization and integration of other techniques like 3D bioprinting are inevitable to reach highly predictive in vitro models even capable of serving as alternatives for animal or (pre)clinical experiments.

## Author Contributions

C. Huang, contributed to design, data acquisition and analysis, drafted the manuscript; F. Sanaei, contributed to data interpretation, drafted and critically revised the manuscript; W.P.R. Verdurmen, W. Ji, contributed to design, data analysis, critically revised the manuscript; F. Yang, contributed to design, data interpretation, critically revised the manuscript; X.F. Walboomers, contributed to conception, data analysis and interpretation, critically revised the manuscript. All authors gave final approval and agree to be accountable for all aspects of the work.
